# Genome Sequencing Reveals Unique Mutations in Characteristic Metabolic Pathways and the Transfer of Virulence Genes between *V. mimicus* and *V. cholerae*

**DOI:** 10.1371/journal.pone.0021299

**Published:** 2011-06-22

**Authors:** Duochun Wang, Haiyin Wang, Yanyan Zhou, Qiuxiang Zhang, Fanfei Zhang, Pengcheng Du, Shujing Wang, Chen Chen, Biao Kan

**Affiliations:** 1 National Institute for Communicable Disease Control and Prevention, Center for Disease Control and Prevention / State Key Laboratory for Infectious Disease Prevention and Control, Beijing, China; 2 Center for Diseases Control and Prevention of Shanxi Province, Taiyuan, China; University of Louisville, United States of America

## Abstract

*Vibrio mimicus,* the species most similar to *V. cholerae*, is a microbe present in the natural environmental and sometimes causes diarrhea and internal infections in humans. It shows similar phenotypes to *V. cholerae* but differs in some biochemical characteristics. The molecular mechanisms underlying the differences in biochemical metabolism between *V. mimicus* and *V. cholerae* are currently unclear. Several *V. mimicus* isolates have been found that carry cholera toxin genes (*ctxAB*) and cause cholera-like diarrhea in humans. Here, the genome of the *V. mimicus* isolate SX-4, which carries an intact CTX element, was sequenced and annotated. Analysis of its genome, together with those of other *Vibrio* species, revealed extensive differences within the *Vibrionaceae*. Common mutations in gene clusters involved in three biochemical metabolism pathways that are used for discrimination between *V. mimicus* and *V. cholerae* were found in *V. mimicus* strains. We also constructed detailed genomic structures and evolution maps for the general types of genomic drift associated with pathogenic characters in polysaccharides, CTX elements and toxin co-regulated pilus (TCP) gene clusters. Overall, the whole-genome sequencing of the *V. mimicus* strain carrying the cholera toxin gene provides detailed information for understanding genomic differences among *Vibrio* spp. *V. mimicus* has a large number of diverse gene and nucleotide differences from its nearest neighbor, *V. cholerae*. The observed mutations in the characteristic metabolism pathways may indicate different adaptations to different niches for these species and may be caused by ancient events in evolution before the divergence of *V. cholerae* and *V. mimicus*. Horizontal transfers of virulence-related genes from an uncommon clone of *V. cholerae*, rather than the seventh pandemic strains, have generated the pathogenic *V. mimicus* strain carrying cholera toxin genes.

## Introduction

*Vibrio mimicus* occasionally causes sporadic diarrhea and extraintestinal infections [1]. Although it was previously recognized as a biotype of *V. cholerae*, it has now been reclassified as an independent species because of differences in a number of biochemical characteristics; e.g., *V. mimicus* is negative for sucrose fermentation, Voges-Proskauer, lipase (corn oil) activity, and Jordan tartrate reactions [2]. However, in terms of pathogenesis, *V. mimicus* and *V. cholerae* are similar due to sharing virulence factors, such as enterotoxins or hemolysins [3], [4], [5], [6], [7]. Although *V. mimicus* has not yet been reported to produce a severe epidemic of diarrhea, it is often isolated from sporadic diarrheal patients, sea water and food, indicating that it may be a potential source of the emergence of a new pathogen, as increasing numbers of genetic elements and virulence factors are exchanged by acquisition of foreign DNA from *V. cholerae* or other bacteria [8].

The naturally occurring strain of *V. mimicus* lives in aquatic ecosystems, and its hosts are phytoplankton and crustaceans [9]. A number of virulence factors have been reported related to human infections [10]. *V. mimicus* is known to produce three types of hemolysins [11]. *V. mimicus* hemolysin (VMH) is heat labile and immunologically similar to *V. cholerae* El Tor hemolysin [5]. Vm-TDH is a heat stable hemolysin that is closely related to the thermostable direct hemolysin (TDH) produced by *V. parahaemolyticus* [6]. Finally, *V. mimicus* produces a novel hemolysin, designated HLX, but little is known regarding its function [11]. Additionally, some clinical isolates of *V. mimicus* have been found to carry a heat-stable enterotoxin (ST) gene identical to that of *V. cholerae* non-O1/non-O139 (nag-st) [4]. However, the determinants of the virulence factors of *V. mimicus* and the molecular mechanisms of the differences in biochemical metabolism between *V. mimicus* and *V. cholerae*, have not been well characterized.

The CTX element, which encodes cholera toxin (CT) in *V. cholerae* [12], is a virulence gene that is well known to be associated with horizontal transfer of the filamentous bacteriophage CTXФ. This element is widely found in epidemic *V. cholerae* but is rarely seen in environmental non-O1/non-O139 isolates [13]. It has been reported that *V. mimicus* isolates from the natural environment can be infected by CTXФ experimentally [14], and four clinical strains, from Bangladesh, India, Japan and the United States, were also reported to carry CTX [8].The receptor for CTXФ, toxin-coregulated pilus (TCP), which is encoded by the vibrio pathogenicity island (VPI) [15], was also present in these *V. mimicus* isolates [8]. This suggests that *V. cholerae* is not the only host of CTXФ and that contemporary horizontal transfer between species has occurred [16]. The potential ability of such strains to cause an epidemic is unclear, but this threat may rise.

Four draft genomic sequences of *V. mimicus* isolates have recently become available, which were produced using the Roche-454 pyrosequencing technology, including strains VM603 (GenBank: NZ_ACYU00000000), VM573 (NZ_ACYV00000000), MB-451 (NZ_ADAF00000000) and VM223 (NZ_ADAJ00000000) [17]. Additionally, 36 published genomes of *V. cholerae* have also been widely studied. Extensive genomic sequencing of *V. mimicus* isolates may increase our understanding of the taxonomy and ecology, as well as the pathogenicity of *V. mimicus*. In this study, we analyzed the variation dynamics of a *V. mimicus* genome carrying the CTX element based on comparative genome analysis with *V. cholerae*. This provided a global view of the genome of *V. mimicus* and a clear map of the phylogenetic relationships with other *Vibrio* spp. Additionally, Instead of making pairwise comparisons among different species, the variations identified were further examined using a combinatorial approach and confirmed for available *V. mimicus* strains. The obtained data provided detailed information of a close relationship and potential horizontal gene transfer between *V. mimicus* and *V. cholerae*, as well as showing the genomic mutations of metabolic pathways used for the identification of these two species.

## Results

### Genome features of *V. mimicus* strain SX-4

**Figure 1:**
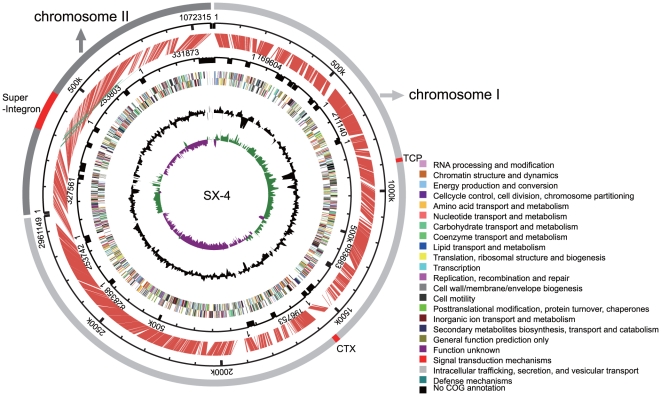
Schematic circular diagram of the *V. mimicus* strain SX-4 genome and synteny relationships between SX-4 and *V. cholerae* strain N16961. Key for the circular diagram (outer to inner): (1) reference genome of *V. cholerae* N16961 and the backbone of our genome as well as their alignment with red lines. (2) SX-4 COG categories on the forward strain (+) and the reverse strand (-). (3) G+C content and GC skew (G-C/G+C) of SX-4 respectively with a window size of 10 kb. Superintegron, CTX and TCP were marked in the figure.

We sequenced the genome of *V. mimicus* strain SX-4 with whole genome shotgun method and the total genome sequences is estimated to 4,391,932 bp (Text S1). 51 IS elements, 102 tRNA, and 25 rRNA clusters were found in our genome. The general features of the genome were summarized in Table 1 and Figure 1.

**Table 1:** General feature of the *V. mimicus* SX-4 genome.

	N16691	Simulated N16961	SX-4	Complete SX-4 (Simulated)
**Genome**				
Size	4033464	3924560	4273349	4391931.67
Contig Number (>1K)	-	57	35	-
N50	-	335321	253803	-
G+C percentage	47.49	47.5	46.25	-
Scaffold Number (1K)	2	54	31	-
Paired read coverage (#)	-	73.65 (3905736)	69.86 (4034050)	-
Single read coverage (#)	-	66.11 (3546891)	61.14 (3572283)	-
Unmap read coverage(#)	-	2.91 (153706)	80.67 (4617101)	-
Aver. Gap size	-	39.33	88	-
				
**Predicted CDS**				
CDS Numbers	3836	3558	3903	4207.96
Average Size	914.94	961.6	960.49	913.88
Percentage of Coding	87.01%	87.18%	87.72%	0.88
Truncated CDS	-	351	384	-

Based on our genome, we got 3903 predicted CDS in *V. mimicus*, among which152 did not return a significant match to the protein database in BLAST searches, indicating potentially unique *V. mimicus* proteins. *V. mimicus* shared 2490 common genes with *V. cholerae*, and these genes are associated with 2332 functions. Genes from the super-integron of *V. cholerae* have also been identified in strain SX-4, as well as some important gene clusters, including those of the O-antigen, hemolysin, CTX, TCP pathogenic islands, polysaccharide complex protein and general metabolism pathway-associated genes, which allow this strain to present a similar pathogenicity to *V. cholerae*. The *Vibrio* seventh pandemic island-II (VSPII) gene cluster, which was only found in the 7th pandemic strains of cholera, was not found in SX-4. Hemolysin represents a potential virulence factor family; in our study, we identified three typical genes, *vmh, tdh,* and *hlx*, in a hemolysin gene cluster, as well as an additional hemolysin-associated gene, *tlh* (thermolabile hemolysin). The *tlh* gene has also been identified in other *Vibrio* spp., such as *V. parahaemolyticus*, which suggests that *V. mimicus* is a potential epidemic pathogen, similar to other *Vibrio* species.

### Genome-based evolutionary analysis showed that *V. mimicus* has a large number of mutations compared to its nearest neighbor, *V. cholerae*

**Figure 2:**
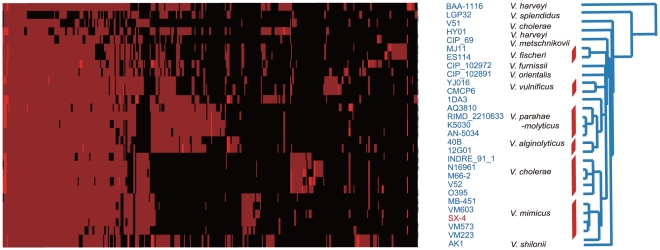
Phylogenetic tree of *Vibrio*. A cluster of 8916 orthologues grouped into 28 strains was screened for the presence (red) or absence (black) of genes. A phylogenetic tree was constructed for all strains, and the red line region represents the same category. On the right nucleotide sequences of homologs were used to construct a phylogenetic tree.

In this study, we intended to shed light on the relationships among *Vibrio* species that are important for the establishment and maintenance of the taxonomy of these species, as well as to construct an identification and diagnosis system. Previously, genomic sequences for *V. mimicus*, *V. alginolyticus, V. fluvailis,* and *V. vurnissii,* as well as *V. cholerae* were obtained using a whole-genome shotgun approach (summarized in Table S1). Phylogenetic analysis based on ∼8,872 kb of orthologous protein-coding regions of 29 *Vibrio* strains revealed that *V. mimicus* had recently diverged from the other species, and that *V. cholerae* was the closest species to *V. mimicus* (Figure 2). With the exception of strains 1DA3, BAA1116, and HY01, which belong to *V. harveyi*, the other isolates belong to different species, as determined based on the recently determined genetic diversity of *Vibrionaceae*. The four newly sequenced *V. mimicus* genomes (MB-451, VM223, VM573 and VM603 [18]) were used for subsequent analysis. In addition to SX-4, all *V. mimicus* strains revealed a similar genome size, GC content, and number of predicted genes (See Table S2).

The entire set of homologous genes of the *V. mimicus* strains was compared in all possible pairwise combinations using BLAST analysis. The percent identity between pairs ranged from 23 to 100%, with no particular bias among *Vibrio* species, except *V. cholerae*. The results of all permutations of the genes and the gene sequences together indicated the relationships among the *Vibrio* species. Comparative analysis of the strains showed that *V. mimicus* shares a higher similarity of genes to *V. cholerae* than do the strains from the other two species (Figure 1). This is similar to the results of previous studies [17], which places *V. mimicus* and *V. cholerae* as the nearest neighbors based on comparative genome analysis results. Consistent with this, the global genome comparisons at the nucleotide level show that 2025 gene families were conserved in these two species, with 2111 and 2802 genes in *V. cholerae* and *V. mimicus*, respectively.

When we mapped the sequenced reads to all of the *Vibrio* genomes, the plot of the similarity was fitted well by the phylogenetic tree, but the percentage of mapped reads showed a large divergence between *V. mimicus* and *V. cholerae* (4.5%∼5.6%). Even among the *V. mimicus* strains, the 52.5%, 63.9%, 64.5% and 97.8% high quality *V. mimicus* reads mapped to VM223, VM603, MB-451 and VM573, indicating that *V. mimicus* has a large number of single nucleotide mutations among its strains with more than a 2% difference, considering that genome matching tolerated 2 bp of mutations per read.

To investigate the divergence between *V. mimicus* and *V. cholerae* with respect their basic life-cyle and metabolism, genes belonging to core gene families, classified according to their predicted functions, were used to construct the core genome carrying housekeeping functions and to perform comparative analysis. The sequenced *V. cholerae* and *V. mimicus* genomes revealed a high level of similarity with respect to homologous genes, which are clustered into 3201 homologous gene families (HGF). Among these HGF, 1014 belonged only to *V. mimicus*, while 323 belonged only to *V. cholerae*. These HGF proteins were mostly involved in essential functions related to the vegetative functions of the bacteria, such as DNA replication and repair elements, protein synthesis and carbohydrate metabolism, as well as fundamental functional categories, such as cell wall synthesis and protein folding (Figure S1).

It has been reported that the two sequenced *V. mimicus* genomes (strains VM573 and VM603) are closest to that of *V. cholerae* El Tor strain N16961[18]. Here, we also compared the genomic content of *V. mimicus* SX-4 with N16961 to reveal the genetic basis for the functional distinction of the bacteria. The isolates shared a high degree of HGF synteny, but we found a great deal of divergence in their genetic variants, indicating these are early divergence strains, although their phenotype and evolutionary distance are close. We found 1479 more genes in *V. mimicus* than in N16961. The chromosomal genes were categorized into common genes and specific genes, as compared to N16961. The analysis clearly identified monophyletic clades that appeared to be lineage specific with ancestral support (Figure S1). These different genes were present or absent in each category and played similar roles in metabolism with similar functions; therefore, this represents a possible reason that the two species show similar biological characteristics and adaptations to their natural environments.

### The characteristic biochemical test differences used in the species identification of *V. mimicus* and *V. cholerae* are mainly caused by gene deletion

*V. mimicus* is very similar to *V. cholerae* in its phenotype, though biochemical tests can be used to discriminate between the two species for the purpose of identification, including a sucrose fermentation test, Voges-Proskauer test and lipase activity test [2]. In contrast to *V. cholerae, V. mimicus* shows negative results for these three tests. A comprehensive analysis of genomes and pathways revealed a deletion of one or more genes that was consistently different between SX-4 and *V. cholerae* N16961 in each of these pathways (Table S3). To investigate the timing of these deletions, we also compared all of the 50 (Table S3) genes to the associated regions in the genomes described in the literature; these sites were found to be accurate and consistent with each other.

**Figure 3:**
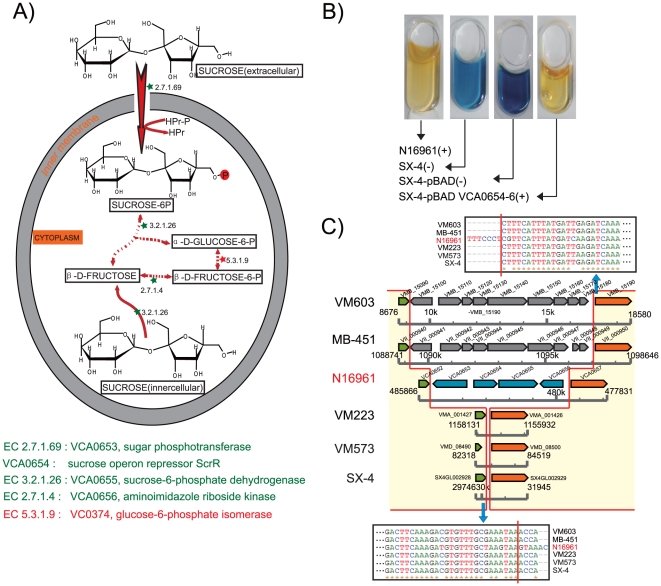
Sucrose fermentation of SX-4 and SX-4-pBADVCA0654-6. (A) Sucrose fermentation in *V. mimicus*; the red portion shows the function of the enzyme translated from VCA0654 to VCA0656 in the pathway. (B) Complement test. The gene cluster of VCA0654 to VCA0656 from *V. cholerae* strain N16961 was complemented into the *V. mimicus* strain SX-4. The strain was grown under LB in the presence of 0.1% arabinose, and then tested for sucrose fermentation (API-20E). Only the strain bearing plasmid pBADVCA0654-6 presented a positive result, like that of *V. cholerae* N16961. (C) Genome structure of the VCA0654-VCA0655-VCA0656 operon. Positions and orientations of ORFs are indicated with different color arrows. Homologue genes are presented with the same color. Homologue regions have been showed with red background, also with their flanking sequences. Insertions in VM603 and MB-451 are homologues presenting with the same color.

The sucrose fermentation test is associated with ability to metabolize starch and sucrose (Figure 3A). In *V. cholerae* N16961, 19 genes are included in this pathway according to the KEGG database (Table S3). In strain SX-4, we found 16 of these genes on the chromosome, with three genes being absent (VCA0654, VCA0655 and VCA0656 corresponding to N16961 genome). A complementation experiment of these genes in strain SX-4 showed that the complementary strain presented similar positive results for sucrose fermentation to what is seen in *V. cholerae* N16961 (Figure 3B), indicating that VCA0654, VCA0655 and VCA0656 (sucrose operon repressor *ScrR,* sucrose-6-phosphate dehydrogenase and fructokinase) are responsible for the differences in sucrose metabolism observed in *V. cholerae*, and their deletion in *V. mimicus* strain SX-4 causes negative sucrose fermentation. We further investigated the available genome sequences of *V. mimicus* strains VM223, VM603, MB-451 and VM573 for these genes. For strains VM223 and VM573, a quite similar deletion of these genes was found (Figure 3C), suggesting this deletion probably occurred before the separation of *V. cholerae* and *V. mimicus*. However, an additional genome fragment insertion was identified in VM603 and MB-451, indicating that the split site is also an active indel region in the genome.

In addition to the sucrose fermentation pathway, the genes (and all of the operons) associated with the other key biochemical tests used for species identification, the Voges-Proskauer test and lipase activity test, were also identified in *V. cholerae* and *V. mimicus*. VC1590, encoding acetolactate synthase, which catalyses the transformation of acetymethyl carbinol into 2,3-bytaylene cylycol in the *V. cholerae* N16961 genome, was lost in *V. mimicus* SX-4, as well as strains MB-451, VM223, VM603 and VM573. The lack of this gene may be reflected in a defect in the catabolism of acetyl-lactic acid and cause the negative result in the Voges-Proskauer test. The lipase activity test is used to detect the pathway for catalyzing and hydrolyzing lipids, forming fat acids, glycerin and monoglycerides or diacylglycerol. In *V. cholerae*, six genes are associated with lipase metabolism, which encode the putative esterase/lipase YbfF (VC2097), lactonizing lipase (VCA0221), a putative lipase activator protein (VCA0222), a lipase of the GDXG family (VCA0490), a lipase-related protein (VCA0754) and a putative lipase (VCA0863). VCA0221 and VCA0222 were found to be absent in *V. mimicus*, which is probably the reason for the difference between *V. mimicus* and *V. cholerae* with respect to the lipase reaction.

### The pathogenic characteristics of *V. mimicus* are highly associated with lateral gene transfer

#### 1. Polysaccharides and lipopolysaccharides

**Figure 4:**
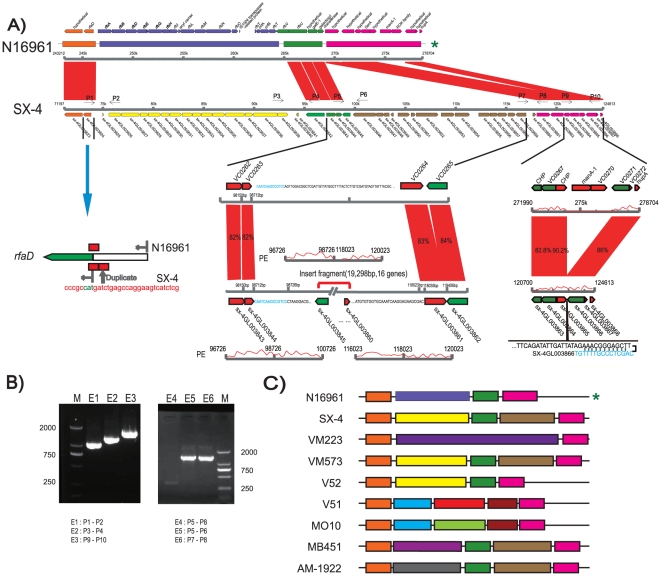
Comparison of the O-antigen biosynthesis cluster from *V. mimicus* strain SX-4 and *V. cholerae* strain N16961. (A) The gene clusters are presented according to the chromosome with different color arrows. The colors of arrows are different units in genome rearrangement between *V. mimicus* and *V. cholerae*. P1 to P10, with black arrows, indicate primers designed to validate the structure. The left bottom figure shows the duplication in SX-4 in this region. The middle bottom figure presents the region and structure associated with genome exchange, as well as the flank. The red curve represents the pair-ends sequence relationship. The right bottom figure shows the downstream region of chromosome with a hairpin structure. (B) PCR results for structure validation. Primers were designed at the location marked in Figure 4A. (C) Scheme of clustering in other available genomes. For better describe the pattern of genome rearrangement in O-antigen region, the cluster were divided into different parts according to their similarity. The similar regions were showed with the same colors, and genes presented with the color bar are consistent with those in Figure 4A. * N16961 has been used as reference genome. PE curve is drawn by supporting pair-ends SOLEXA reads coverage.

In *V. cholerae*, the polysaccharide gene cluster is flanked by *rfaD* (encoding D-glycero-D-manno-heptose 1-phosphate guanosyltransferase, which is involved in lipopolysaccharide core biosynthesis), and *rjg* (encoding a conserved hypothetical protein with similarities to the mRNA 3′-end processing factor) [19]. However, the structure of this cluster in *V. mimicus* is unclear. Fortunately, in SX-4, a similar clustered organization of its genes was observed, which contained a backbone of two genes, SX-4GL003824 (homolog to *rfaD*) and SX-4GL003861 (homolog to *rjg*) (Figure 4A). Between these two genes, SX-4 contains a ∼22 kb specific antigen encoding region, which differ to that of *V. cholerae* O1 and O139 (Figure S2). This region contained 17 genes and one IS element (Figure 4A), following 13 bp of repeat sequences in the *rfaD* gene, through the end of the *rfb* cluster (*rfbU*), which were predicted to belong to the O37 antigen cluster-associated genes in *V. cholerae* V52 and MZO-3, encoding enzymes or proteins of unknown function, similar to *Rickettsia bellii* OSU 85-389. In another conserved end region, we found a ∼19 kb insertion between VC0263 and VC0264, corresponding to the N16961 genome, encoding 16 genes in strain SX-4, the majority of which were specific and hypothetical genes. However, two loci were identified in the cluster that were similar to the bacterial sugar transferase and *wca* protein in *Aggregatibacter aphrophilus* NJ8700, indicating that the region was part of the O-antigen or a complementary region. The following two conserved genes in N16961 have a hairpin structure (AAACGGGAGCT-TC-AGCTCCCGTTT) in SX-4, causing three genes deleted compared to *V. cholerae* N16961 (Figure 4A). It appears that the polysaccharide produced by SX-4 has a different structure than that of N16961. Therefore, this complex mosaic structure of polysaccharides is likely to have been caused by genomic exchanges between different species.

Genetic exchange in the ‘O’ antigen synthesis region (Figure 4A) has been widely reported in *V. cholerae* and plays important roles in the evolution of many pathogenic bacteria, as shown for the O139 cholera outbreak [20], [21]. Analysis of the schema structure in this region from four additional available draft genomes of *V. mimicus* suggested that the six genomes might encode different polysaccharides and present different serotypes, except for VM573, which was similar to the SX-4 strain sequenced here (Figure 4B,4C, Figure S2). Notably, V52, which is a serogroup O37 *V. cholerae* strain, presented a similar structure to SX-4 at the beginning of the conserved region, apart from the above mentioned component region at the end of this region, it showed a similar structure to N16961. These opposite divergences in strain V52 showed that the insertion at the end of the conserved region might not be necessary for the bacteria but may complement a better-constructed surface protein, indicating that the mosaic structure of this region may be generated by DNA shuffling individually.

#### 2. CTX element

**Figure 5:**
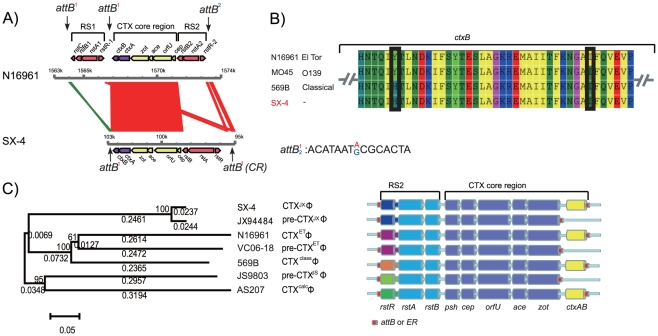
CTXΦ alleles and the phylogenetic tree for different strains of serogroups and biotypes. (A) The CTX gene cluster compared to its associated region between SX-4 and N16961, as well as their flanking sequences. (B) Partial *ctxB* sequences show different mutations among the O1 and O139 serogroups. (C) The phylogenetic tree based on alignment results of *rstR-ig2* sequences from the seven typical strains. The right portion is the modeled map for different types of CTXФ genomes.

The highly invasive characteristics of *V. cholerae* that cause fulminate diarrhea in humans are associated with the CT that resides in epidemic *V. cholerae* isolates. Strain SX-4 was isolated from a watery diarrheal patient and carries *ctxAB*. By the real-time PCR method [22] and genome analysis, we found SX-4 contains a single copy and a typical genetic organization of the CTX element, like that of *V. cholerae* N16961, but no RS1 was found (Figure 5A). This result is consistent with the validation with the SOLEXA reads coverage in the genome. Also, the flanking sequences of CTX element in SX-4 are highly homologue to that of *V. cholerae* N16961 chromosome I, suggesting that the CTX element in SX-4 are potentially located in large chromosome. The element contains the typical RS2 and core regions of CTX. In *V. cholerae,* the core region plays a vital role in the morphogenesis of CTXФ, and the RS2 region is necessary for integration, replication and regulation of the CTX prophage genome. This suggests that SX-4 is able to be infected by CTXФ through its lysogenic conversion mechanism and can cross species. Recently, Kumar et al [23] found a new type of mutation in *ctxB*. We investigated this gene sequence and observed the same sequence as in the classical biotype sequence of *ctxB* (Figure 5B).

*rstR*, identified as a biomarker for CTXФ in the RS region, divides the CTX element into different alleles in the classic and El Tor biotypes, which are CTX^class^Ф, CTX^ET^Ф, CTX^calc^Ф, and their pre-phages, as well as other special types, such as pre-CTX^JS^Ф (AF302794) and pre-CTX^JX^Ф (AF511002). Other alleles, including rstR4*, rstR4**, *rstR5*, *rstR6* and *rstR232* from O1/O139 and non-O1/non-O139 strains, have been reported [24], [25], [26]. For strain SX-4, sequence comparison showed its *rstR* has high similarity to that of the O139 strain JX94484 (Figure 5C), which is also known as rstR4** [26]. However, these environmental non-O1/non-O139 strains and the O139 strain JX94484 possess pre-CTXФ. Based on the *rstR* and *ctxB* sequences, it may show that CTX prophage on SX-4 genome was not obtained from the common 7th pandemic El Tor clones which carrying El Tor type *ctxB* and El Tor type *rstR* genes in CTX element.

In *V. cholerae*, two important toxin-associated components, TLC and the RTX cluster, locate on the flanks of the CTX element [27], [28]. Commonly, TLC has been found to be located in the 5′ region of the CTX gene cluster and is associated with CTX cluster integration, while the RTX cluster is located in the 3′ region of CTX. Both of these elements were absent in *V. mimicus* SX-4, as well as in MB-451 and VM223, which are also CTX prophage-positive strains. These data suggest that it might not be strictly necessary for CTX and TLC to co-occur in *V. mimicus*, or in *V. cholerae*.

In SX-4, the CTX element integrated into the chromosome via the characteristic attachment site (*attB*) on the flank of the CTX element (Figure 5A). The 5′-end *attB* sequence harbored additional pseudogenes, which probably encodes a para-aminobenzoate synthase glutamine amidotransferase component II protein, similar to *V. cholerae* V52 (gb|EAX60338.1|), whereas the 3′-end *attB* is in the intergenic regions associated with recognition sites for insertion. This *attB* is the same as attB^1^ in N16961, but differ with 1 bp to attB^2^ in N16961 (Figure 5A).

#### 3. TCP pathogenicity island

TCP, the specific receptor of CTXФ in *V. cholerae,* is an integral part of the VPI. Analysis of the complete sequence of SX-4 identified all of the TCP genes typically found in *Vibrio* species, with a similarity of 87∼100% for each gene. When compared to the El Tor strain N16961, a putative att-like 20-bp attachment site on the flank of the TCP gene cluster, including complete gene clusters, such as *tcpI, toxR, toxT* and *tagA-tagD*, suggested interference with microbial attachment to host tissues. Additionally, among *V. cholerae* isolates, the sequence of the *tcpA* locus is more divergent compared to other loci in the VPI. Most likely, this diversity is a reflection of diversifying selection during adaptation to the host immune response or to CTX susceptibility [29]. TCP is a receptor used by the CTX phage to infect *V. cholerae*, except for in a few strains with the CTX element but no *tcp* genes [29]. Therefore, in our analysis, the *tcpA* gene was used to construct a phylogenetic tree of the *V. cholerae* cluster for a CTX acquisition event that occurred in a TCP-dependent manner; the *tcpA* gene of SX-4 is identical to that of the *V. cholerae* serogroup O115 strain [25].

## Discussion

In this study, we sequenced the genome of a *V. mimicus* strain carrying an intact CTX element and compared it with the available genomes of other *Vibrio* spp. Analysis of the phylogenetic relationships among these species provided important insight into the genomic history related to the *Vibrio* genus and the rules for evaluating *Vibrio* evolutionary distances. Our experimental results and computational analysis, along with evolutionary clues and bacterial phenotype coupling among homologous genes, suggest that the genomes of *V. mimicus* are quite different from the pandemic *V. cholerae* strains. Considering the complexity of the serogroup structure of *V. cholerae*, genome sequencing in other serogroups of *V. cholerae* may help to reconstruct more accurate evolutionary relationships between *V. mimicus* and *V. cholerae*.

Species identification is commonly based on biochemical tests in *V. cholerae* and *V. mimicus*. Our analysis of the key biochemical test-related differences in the genomes of these species revealed the molecular mechanisms underlying the biochemical test differences between them and provided clues to link the genome features and classical biochemical tests. Based on operon structures, we uncovered the deletion of gene sequences related to these three biochemical tests. All of the operons shared clear insertion sites, which indicates that the differences related to these biochemical tests are caused by ancient events in evolution that took place before the divergence of *V. cholerae* and *V. mimicus*. It is interesting that there are different modes of the mutation of the gene cluster involved in the sucrose fermentation in different *V. mimicus* strains, including insertion and deletion. However, why sucrose utilization is prevented in *V. mimicus* is unknown. It could represent an important event in the evolution of *V. mimicus* that benefited these strains in adapting to a special micro-ecosystem in a particular period.

In this study we sequenced the genome of a *V. mimicus* isolate carrying an intact CTX element from a cholera-like patient. Strain SX-4 also carries a number of hemolysin genes, including the *vmh*, *tdh*, *hlx* and *tlh* genes, which indicates a possible pathogenic character. Additionally, this strain carries CTX, which integrated into its chromosome through an *attB* site, similar to what is seen in the toxigenic *V. cholerae*. Notably, this strain carries the rstR4** type of CTXФ, which has also been found in an O139 serogroup strain (our unpublished data) and non-O1/non-O139 strains [26]. In these *V. cholerae* strains pre-CTXФ prophage carrying rstR4** were found, whereas in *V. mimicus* SX-4, it is the intact genome of CTXФ carrying rstR4**. It can be assumed that the intact CTXФ prophage genome in SX-4 was obtained from a *V. cholerae* strain carrying such an element. Additionally, the *ctxB* gene in SX-4 is of the classical type, which was found in strains of the classical biotype worldwide and in U.S. gulf coast strains [30], Based on these two characters of its CTX prophage genome, strain SX-4 may not be transferred from the typical 7th pandemic El Tor strains, but rather, from a different toxigenic clone. These data also show the abundance of this lysogenic phage and its active transference within *V. cholerae* and its sister species.

Strain SX-4 also includes the TCP island, suggesting a closer relationship to the toxigenic species *V. cholerae* and that it in terms of colonizing and pathogenic characteristics compared to other *V. mimicus* strains. The *tcpA* sequence of SX-4 is identical to that of a *V. cholerae* serogroup O115 strain [25], but not the classical or El Tor type. Additionally, genome comparison showed that the LPS gene cluster is expected to have been recombined in *Vibrio* genome evolution. These islands have been proven to be associated with virulence [31], [32], [33], [34]. Here, we provide more detailed genomic information related to their exchange among different *Vibrio* spp. The sequence identity of both the CTX and VPI genes derived from *V. mimicus* and *V. cholerae* strongly suggests that successive horizontal transfer of bacteriophages from different clones or serogroups to *V. mimicus* has occurred.

In summary, we exploited genome information to investigate the properties of a *V. mimicus* virulent strain, SX-4, and the evolutionary relationships among *Vibrio* spp. Horizontal gene transfer may be proposed to be common between *V. mimicus* and *V. cholerae*, especially for virulence-related genes. In *V. mimicus*, the common gene mutations observed in a number of metabolic pathways related to the biochemical tests used for species identification may suggest different adaptations and evolution tendency in these species. The SX-4 isolate described in this study is not the only strain of *V. mimicus* carrying CTX that can cause diarrheal disease in humans, but the evidence presented here indicates that this may be forming a potential environmental reservoir for CTXФ and other virulence factors and may play an important role in the emergence of new toxigenic clones. It may also suggest that caution is necessary regarding a possible epidemic caused by *V. mimicus* strains carrying cholera toxin genes.

## Materials and Methods

### Strains and culture conditions

*V. mimicus* strain SX-4, isolated from a 55-year-old male patient who suffered from cholera-like diarrhea in 2009 in Shanxi, China, was used to construct shotgun libraries for genomic sequencing. According to a number of biochemical characteristics, including being negative for sucrose fermentation, Voges-Proskauer, and lipase tests, combined with the results of the API20E/NE test, the isolate was confirmed to be *V. mimicus*. PCR was used to test the *V. mimicus* SX-4 isolate for the presence of *ctxA* and then confirmed again with the genome sequencing.

SX-4 was routinely cultured using Luria-Bertani (LB) medium at 37°C. Genomic DNA was extracted from a 10-ml overnight culture using the TIANamp Bacteria DNA Kit (TIANGEN Biotech, Beijing, China). Genomic DNA was quantified on a 0.7% agarose gel, stained with ethidium bromide and spectrophotometrically assessed. The stock DNA solution was separated into two aliquots, one of which was used for sequencing, while the other was stored at −20°C for further PCR gap closing and structure validation.

The other genomic sequences used in this study were obtained from GenBank. The strain names and their accession numbers are summarized in Table S1.

### DNA sequencing and annotation

An initial *V. mimicus* shotgun genome sequence (accession number. ADOO01000000) was obtained from a mixture of Solexa sequences from pair-ends Solexa sequencing of a 500-bp insert size library to 130X coverage. The draft genome sequence was assembled into contigs using the novel short-read assembler SOAPdenovo [35]. The Solexa library produced a total of 4,034,252 reads, providing ∼63-fold sequences coverage and was *de novo* assembled into 35 contigs, of which 4 contigs were linked by paired ends, with contigs N50 253,803 bp. The 39 contigs were aligned to the whole genome sequence of *V. cholerae* to identify the gaps to be sequenced in the genome of *V. mimicus*. All genome structure variations have been carefully checked to avoid assemble errors, the prediction was verified by PCR methods. The genomic structure of CTX element and major virulence factor mutations in SX-4 was one of the main aim in this study, although we did not close all of the gaps in the genome and determine the order of the contigs, we closed all of the gaps in three major lateral gene transfer regions, as well as the potential virulence genes by PCR walking. Amplicons were obtained by PCR using the genomic DNA of strain SX-4 as the template and sequenced. These new sequences, together with the contigs will be used to determine the whole-genome sequence in future analyses.

Putative CDS were identified by the Glimmer gene prediction program [36], and their functions were automatically annotated using BLASTP (e-value <1e-10 identity>80 and >100 aa) against GenBank and UniProt (version 47). For each CDS, the ORF sequences were further categorized based on Interpro, GO, and COG (Clusters of Orthologous Genes). Functional pathways were annotated based on KEGG. tRNA genes and repeats were predicted with tRNAscan-SE and Repeatmasker [37], respectively. The genome sequences of *V. mimicus* have been deposited in GenBank.

### Comparative genome analysis and phylogenetic tree construction

Twenty-seven genomes of Vibrionaceae species available from GenBank (Table S1), representing 14 different species, were used in our analysis. Homologous genes were identified by comparisons using OrthoMCL [38] with BLASTP (e-value <1e-5) at the genome level. The genomes were automatically compared for missed gene calls by *Perl* scripts based on homolog relationships. Housekeeping genes were used in the construction of the phylogenetic trees for *Vibrio* by the neighbor-joint method in Paup [39]. Consistent with this, the homologous genes in each complete genome were clustered using a hierarchical cluster method. Their nucleotide sequences were also used to uncover evolutionary clues by Paup. The nucleotide alignment of the *tcpA* gene, which occurs widely in different serogroups, and the component of the CT element receptor, which presents the possible evolutionary relationships of the CT element among the different serogroups, then used to construct a phylogenetic tree by the neighbor-joining method in Mega4. The *rstR* gene was also used to construct the phylogenetic tree to specify the source of the CT element in different *V. cholerae* local strains by the neighbor-joining method in Mega4. The sequence alignment of *ctxB* was edited by Mega4.

To identify the potential genes that differ between the CT-carrier strain *V. mimicus* SX-4 and *V. cholerae* N16961, pairwise and reciprocal comparisons were performed by aligning the predicted genes of one strain to the whole-genome sequence of the other and vice-versa. The genome sequences were compared for missing genes, and homologous hits were identified in the second genome. The similar regions were annotated on a synteny line map or circle map with red blocks. Detailed comparative analysis of duplicated sequences and differences between the two strains in their genome structure were performed by Perl scripts. Solexa read copies along the genome in the typical region were calculated using SOAP mapping results. SNPs were searched using the software package Mummer and then annotated to the genome of *V. cholerae.*

### Verification of genome transfer and biochemical pathway complement test

To verify the insertions and deletions in the genome of SX-4, four sets of unique primers were designed to perform a PCR-based examination of the DNA of SX-4. The primers are indicated as arrows in Figure 4A. Routing PCR was carried out with a 50 µl reaction. Amplification was carried out for 30 cycles, with initial denaturation at 95°C for 5 min, denaturation at 95°C for 30 s, annealing at 59°C for 45 s, extension at 72°C for 1 min per kb, and a final step at 72°C for 5 min.

Genes associated with three key biochemical pathways: sucrose fermentation, Voges-Proskauer and lipase (corn oil) activity, were screened and listed according to the KEGG database (http://www.genome.jp/kegg/) (Table S3). All of these genes were aligned to *V. mimicus* with BLAST, with overlap of less than 80%. A set of unique primers was designed to amplify fragments with missing genes (VCA0654, VCA0655 and VCA0656) in SX-4 using *V. cholerae* N16961 as DNA template. The amplified fragments were ligated to the expression vector pBAD24 [40], and the constructed plasmid was introduced into Escherichia coli DH5α, resulting in plasmid pBAD VCA00654-6. The plasmid was then transformed into the SX-4 strain. To induce production of VCA00654-6, the SX-4 strain was grown under LB in the presence of 0.1% arabinose with appropriate antibiotics at 37°C for 4 hours and then tested for sucrose fermentation following manufacturer's instructions (API-20E, bioMerieux, Inc.).

### Copy number determination of CTX element on strain SX-4 genome

The copy number of CTX in SX-4 genome was determined according to a real-time quantitative PCR method we used previously [22] and also SOLEXA pair-ends reads coverage esitmation (Figure 1). And also, we check the assembled sequences linked with CTX element and compared the sequences to the published *V. mimicus* and *V. cholerae* genome using BLAST. In *V. cholerae,* their chomosome contains a single copy of house keeping gene *thyA, *which was used as the reference in our study. The CTX element gene *zot* was used to determine the copy number of CTX. By real-time quantitative PCR, the copy number(CN) of *zot* in different strains was determined with CN = 2^△Ct^ , where *△Ct* means the difference *Ct* value between *thyA* and *zot*. The specific copy number of CTX was obtained according to CN with the integral function. *V. cholerae* O1 strains N16961 (one copy of CTX), GD93284 (two copies of CTX) and 40–42 (zero copy of CTX) have been used as positive and negative control.

## Supporting Information

Text S1 Genome sequencing, assembling and gene content prediction.
(DOC)

Figure S1 Gene function for different categories in *V. mimicus*.
(EPS)

Figure S2 Genome variation in polysaccharides among all available genomes.
(EPS)

Figure S3 Simulation genome and its gap distribution according to *V. cholerae* genome.
(EPS)

Table S1 Genomes of *V. cholerae* and *V. mimicus* used in our studies.
(XLS)

Table S2 Comparative genome size, GC content, and number of predicted genes of *V. mimicus* and *V. cholerae*.
(XLS)

Table S3 Comparative of metabolic gene sets in three key biochemical tests.
(XLS)
