# Supplementary material for: Genome Sequencing Reveals Unique Mutations in Characteristic Metabolic Pathways and the Transfer of Virulence Genes between V. mimicus and V. cholerae
Source: PLoS One. 2011 Jun 22;6(6):e21299. doi: 10.1371/journal.pone.0021299 (PMC3120857; doi:10.1371/journal.pone.0021299)
Supplement: Text S1 — Genome sequencing, assembling and gene content prediction. (DOC) [file pone.0021299.s001.doc]

Estimate the impaction of whole genome shotgun sequences

We sequenced the genome of *V. mimicus* strain SX-4 with a shotgun method using a high throughput Illumina SOLEXA sequencing machine. To better understand the potential gene content included in the genome gaps, we simulated reads of the same quality in the closest complete genome, that of *V. cholerae* N16961, and performed simulated gap analysis after comparison to the *V. cholerae* genome. The simulated genome (SG) gap analysis allowed us to amend genome features, such as genome length, GC content, and gene number. Our assembly spanned a total distance of 3,924,560 bp in the SG, consisting of 108,904 bp, with the remaining 97.3% in captured gaps, although the sequenced data contained >99% of the genome. Thus, we were able to estimate that the genome of *V. mimicus* was ~4,391,932 bp and contained ~4,208 genes. Next, the genomic gaps of *V. mimicus* were examined relative to the *V. cholerae* simulated model to reveal the genetic variation coordinated with a simulated genome (Figure S3). In the SG model, 52 of 69 SG gaps had an average length of 877 bp, and these were duplicated in the genome two or more times by repeat sequences >200 bp. The remaining gaps are all less than 100 bp. The repeat sequences, which are smaller than 100 bp, appeared to have little impact on the genome assembly, so we were able to find short repeat sequences, including some tandem repeats to identify the associated VNTR target for detection. In total, we found 51 IS elements, 102 tRNA, and 25 rRNA clusters. Compared to the SG analysis, 18 IS elements were missing, but no tRNAs or rRNAs were lost. Notably, the super-integron sequences were observed to have been severed by repeats. Finally, previous studies have discovered gap regions enriched with proteins of unknown function, which indicated that the conserved metabolism proteins were reserved in draft genome sequences, whereas some important genes may have been truncated by our assembly. In the SG model, 63 of 80 genes presented unclear functionality, and the remaining virulence-related genes (e.g., *rstA*, *rstB* of CTX element) were found in our assembled sequences.
